# Appropriate Complementary Feeding Practice and Its Associated Factors among Mothers Who Have Children Aged between 6 and 24 Months in Ethiopia: Systematic Review and Meta-Analysis

**DOI:** 10.1155/2022/1548390

**Published:** 2022-09-22

**Authors:** Asrat Hailu Dagne, Shimeles Biru Zewude, Abenezer Melkie Semahegn

**Affiliations:** Department of Midwifery, Debre Tabor University, Debre Tabor, Amhara Region, Ethiopia

## Abstract

**Background:**

Appropriate complementary feeding practices prevent malnutrition among children. The proportion and determinant factors of appropriate complementary feeding practices identified by different studies were inconsistent in Ethiopia. Therefore, this systematic review and meta-analysis aimed to assess the pooled proportion and determinants of appropriate complementary feeding practices among mothers.

**Methods:**

Databases (PubMed, HINARI, Google Scholar, Cochrane Library, and Web of Science) and university repositories were used to search for important articles. A critical appraisal of the studies was conducted. Data analysis was conducted using STATA version 11. Cochran (Q test) and I^2^ test were used to test the heterogeneity of the studies. Publication bias was checked using the funnel plot for asymmetry and Egger's regression test.

**Results:**

Seventeen primary studies with a total sample size of 9166 mothers were involved in this study. The pooled proportion of appropriate complementary feeding practices among mothers who had infants and young children aged between 6 and 24 months was 21.77 (with a 95% CI: 14.07–29.48). Mothers' educational status of secondary school and above (OR = 3.36 with a 95% CI: 3.03–3.69), having repeated antenatal care visits (OR = 4.77 with a 95% CI: 3.49–6.05), child's age between 12 and 24 months (OR = 3.7 with a 95% CI: 2.75–4.65), having repeated postnatal care visits (OR = 3.17 with a 95% CI: 1.96–4.38), health education (OR = 4.88 with a 95% CI: 3.86–5.9), knowledge of mothers (OR = 4.85 with a 95% CI: 3.77–5.93), maternal age between 18 and 35 years (AOR = 2.67 with a 95% CI: 1.64–3.72), institutional delivery (OR = 2.23 with a 95% CI: 1.79–2.68), and higher household wealth (OR = 2.65 with a 95% CI: 1.46–3.84) were found to be statistically significant associated factors of appropriate complementary feeding practices among mothers.

**Conclusions:**

The pooled proportion of appropriate complementary feeding practices was low in Ethiopia. Knowledge of mothers and maternal health service uptake such as antenatal care, postnatal care, and institutional delivery increase appropriate complementary feeding practices. More focus is required for mothers who have children aged less than 12 months, mothers aged above 35 years and less than 18 years, lower mothers' educational status, and lower household wealth. Therefore, integrated interventions are still required to improve appropriate complementary feeding practices.

## 1. Background

Complementary feeding (CF) is started with soft, semisolid, and solid foods and liquids for children aged 6 months when mothers' breast milk alone is insufficient to meet the child's nutritional needs [1, 2]. Appropriate complementary feeding practice (ACFP) is the continuous process of feeding infants and young children considering the minimum required food groups with proper meal frequency based on the age of infants and young children while breastfeeding continues for two years for breastfeeding mothers [3, 4]. ACFP decreases the risk of stunting, wasting, and being underweight. Thus, ACFP prevents malnutrition that reduces the immune system and causes frequent illness in children [5, 6].

Malnutrition is one of the most common problems in the world, particularly in developing countries [7, 8]. There are more than ten million under-five child deaths in the world every year, of which 98% occur in developing countries [9]. Malnutrition has been accountable for 60% of under-five deaths directly or indirectly out of 10.9 million IYC deaths, whereas breast milk (BM) and complementary feeding practices could prevent 13% and 6% of under-five mortality, respectively [2, 10]. Ethiopia is one of the countries where infant and under-five mortality is the highest at 47 per 1000 live births and 59 per 1000 live births, respectively. Moreover, the rates of stunting, being underweight, and wasting among under-five children were 37%, 21%, and 7%, respectively [11].

A crucial window of opportunity is infants and young children (IYC) aged between 6 and 24 months. This window of opportunity is a period to prevent malnutrition, and it is also a period to prevent the long-term adverse outcomes of malnutrition. Therefore, guaranteeing appropriate nutrition during IYC aged between 6 and 24 months is the main priority of global health [12].

The proportion of ACFP is unacceptably low in low-income countries such as 23% in Bangladesh [13], 7.7% in Malawi [5], and 9.76% in Ethiopia [14]. In Ethiopia, the prevalence of ACFP among mothers who had IYC aged between 6 and 24 months was from 8.5 in a study held in Damot Weyde district to 70.3% in a study held in Debre Birhan town [15–31].

Known independent predictors of ACFP are knowledge of mothers on IYC feeding practices, health education on IYC feeding practices, having repeated antenatal care (ANC) visits, having repeated postnatal care (PNC) visits, child age, maternal age, mothers' occupation, workload, mothers' educational status, birthplace, number of children, food secured in the household, and higher household wealth [16, 18, 20, 21, 23–29, 31].

The Ethiopian Federal Ministry of Health in collaboration with other sectors such as the Federal Ministry of Agriculture has developed a national nutrition plan in Ethiopia. The national nutrition plan in Ethiopia embraces ACFP for IYCs aged between 6 and 24 months. Different interventions were carried out to boost the nutritional status of IYC in the country [32]. Even though different interventions were carried out to boost ACFP in Ethiopia, the problem is still high and different from study setting to study setting [15, 16]. Moreover, a national survey of ACFP was also unavailable in Ethiopia.

Comprehensive evidence is lacking concerning the proportion of ACFP and its associated factors among mothers who had IYC aged between 6 and 24 months in Ethiopia. There were great differences and inconsistencies regarding ACFP and its associated factors among mothers who had IYC in the country. The findings of this study could be an input to policymakers, program managers, stakeholders, and health service providers to improve ACFP in this country. Therefore, this systematic review and meta-analysis aimed to determine the pooled proportion of ACFP and to identify the pooled predictors among mothers who had IYC aged between 6 and 24 months in Ethiopia.

## 2. Materials and Methods

### 2.1. Study Design

A systematic review and meta-analysis were engaged to estimate the pooled proportion of appropriate complementary feeding practice and its associated factors among mothers who had infants and young children aged between 6 and 24 months in Ethiopia.

### 2.2. Searching Strategies

Both published and unpublished studies conducted on ACFP and its associated factors among mothers who had IYC aged between 6 and 24 months in Ethiopia were searched. Studies reported in Ethiopia were searched exhaustively by three authors (AHD, SBZ, and AMS) from international databases (PubMed/MEDLINE, HINARI, Google Scholar, Cochrane Library, and Web of Science) and university repositories. University repositories were searched from the website, and articles in the libraries were identified to get unpublished articles through communication with the authors by e-mail. Article search was carried out from December 28, 2021, to January 27, 2022. Search strategies were conducted, and the articles were searched by using search terms (S1 Supplementary Materials) and identifying the full titles on 1/24/2022 to retrieve all important articles.

## 3. Inclusion and Exclusion Criteria

**Figure 1:**
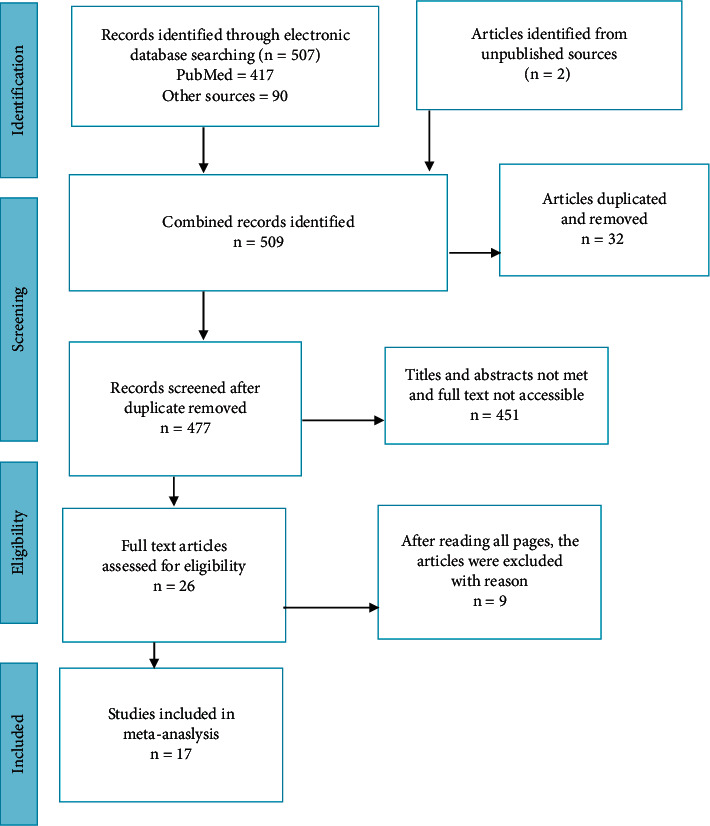
PRISMA flow chart revealing study selection for systematic review and meta-analysis of ACFP among mothers who had IYC in Ethiopia.

Inclusion and exclusion of studies for the systematic review and meta-analysis were presented using the Preferred Reporting Items for Systematic Review and Meta-Analysis (PRISMA) guidelines [33] (Figure 1).

### 3.1. Inclusion Criteria

This systematic review and meta-analysis included studies that meet the following criteria: (1) studies conducted in Ethiopia, (2) studies reporting the proportion and associated factors of ACFP among mothers who had IYC aged between 6 and 24 months, (3) studies published at any time, (4) unpublished studies conducted at any time, and (5) studies that assured the results based on World Health Organization (WHO) IYC feeding indicator definitions of continued breastfeeding, the introduction of solid, semisolid, or soft foods, minimum dietary diversity, minimum meal frequency, and minimum acceptable diet [34].

### 3.2. Exclusion Criteria

In the searching strategy, studies that were not fully obtained from the database and when the corresponding author failed to respond within two weeks of contact by e-mail were excluded. Articles that had only the abstract part and there was impossible to get all other components of the articles were also excluded.

## 4. Measurement of Outcome Variables

The first outcome variable of this study was the proportion of ACFP among mothers who had IYC aged between 6 and 24 months. The second outcome variable of this study was the frequently repeated associated factors of ACFP. The pooled effects of all outcome variables were estimated in the meta-analysis.

### 4.1. Data Quality Assurance

First, all retrieved articles were exported to EndNote 7 reference manager, and duplicate studies were avoided. Three reviewers (AHD, SBZ, and AMS) were coupled, and they independently reviewed the titles and abstracts of the studies to be included in the systematic review and meta-analysis. Then, studies unrelated to the topic of this study, abstracts, and without full text were removed. Finally, studies that met the inclusion criteria were considered. The disagreements on the inclusion and exclusion criteria were resolved through consensus among authors. A critical appraisal of the studies was carried out using the Newcastle Ottawa Quality Assessment Scale (NOS) [35]. The quality of the studies was assessed by two reviewers (AHD) and (SBZ), and the disagreements during the quality assessment were also resolved by a third reviewer (AMS) by evaluating the articles and resolving inconsistencies to reach a consensus. Articles with a quality score of greater than or equal to seven out of ten scores were decided as low risk for bias and considered in the systematic review and meta-analysis (Table 1).

### 4.2. Data Extraction

Data extraction was conducted using a format prepared in the Microsoft Office Excel Worksheet. The data abstraction form was prepared by two authors (AHD and SBZ) to assess ACFP and associated factors among mothers who had IYC aged between 6 and 24 months. The heading of this form has the name of the first author, year of publication, study setting, study design, region, study period, sample size, sampling techniques, the total number of participants, the proportion with the 95% CI, and the standard error of the proportion. Similarly, the heading of the abstraction form had an adjusted odds ratio (AOR) with 95% CI and standard errors of AOR for identified associated factors (i.e., knowledge of the mother on ACFP, repeated ANC visits, food secured in the household, number of children, educational status of mothers, institutional delivery, counseling and advice on ACFP, child age, maternal age, repeated PNC visits, and higher household wealth). The disagreement between the authors (AHD and SBZ) during data extraction was resolved by a third reviewer (AMS) by evaluating the articles and resolving inconsistencies to reach a consensus.

### 4.3. Statistical Analysis

Data were extracted and analyzed using Microsoft Office Worksheet and STATA version 11, respectively. Forest plots with a 95% confidence interval presented the pooled proportion and associated factors of ACFP among mothers who had IYC aged between 6 and 24 months. Moreover, Cochran (Q test) and I^2^ tests were used to identify the random variations between studies [36], and heterogeneity was interpreted based on I^2^ value = 0%, which means no heterogeneity, I^2^ value = 25% means low heterogeneity, I^2^ value = 50% means moderate heterogeneity, and I^2^ value = 75% means high heterogeneity [37]. Publication bias was checked by visual inspection of the funnel plot for asymmetry subjectively, and it was evaluated by using Egger's regression test objectively [38]. Both fixed and random models were used for studies with no heterogeneity and with heterogeneity, respectively. Subgroup analysis was carried out based on sample size, regions, publication year, and community-based and facility-based cross-sectional study design to see the homogeneity and heterogeneity of the proportion of ACFP and its associated factors among subgroups.

## 5. Ethics Approval and Consent to Participate

Since this study was conducted based on data extracted from unpublished and published primary studies, ethical approval was not needed. Moreover, participants' consent was not taken due to data extraction from primary studies.

## 6. Results

A total of 509 articles were identified from electronic databases and unpublished sources. These identified articles were exported to EndNote 7 reference manager, and thirty-two duplicated articles were removed. After reviewing titles and abstracts, 449 articles were excluded due to unrelated to ACFP and associated factors among mothers who had IYC aged between 6 and 24 months. Two articles were excluded due to the inaccessibility of the full text. Nine articles were also excluded due to differences in operation definitions of ACFP, poor, quality, and not reporting outcome variables (the proportion of ACFP). Finally, a total of 17 primary studies were included in this systematic review and meta-analysis.

### 6.1. Characteristics of the Included Articles

A total of 17 primary studies, of which 15 were published, and 2 unpublished studies reported on ACFP and associated factors among mothers who had IYC aged between 6 and 24 months. All included studies were cross-sectional study designs. A total of 9166 mothers who had IYC aged between 6 and 24 months were involved in this systematic review and meta-analysis to estimate the pooled proportion of ACFP of mothers who had IYC aged between 6 and 24 months and to estimate the pooled AOR of predictors of ACFP. Studies conducted from 2014 to 2021 were considered in the systematic review and meta-analysis. The included articles were conducted in 5 regions of Ethiopia. From the 17 articles, 7 [16, 17, 23, 25, 26, 29, 31], 5 [15, 20–22, 28], 3 [19, 24, 30], 1 [27], and 1 [18] studies were conducted in Amhara, SNNPR, Oromia, Tigray, and Afar Region, respectively. The highest proportion of ACFP was reported by the study conducted in Debre Birhan town [16], whereas the lowest proportion was in Damot Weyde district [15] (Table 1).

### 6.2. The Pooled Proportion of Appropriate Complementary Feeding Practices

**Figure 2:**
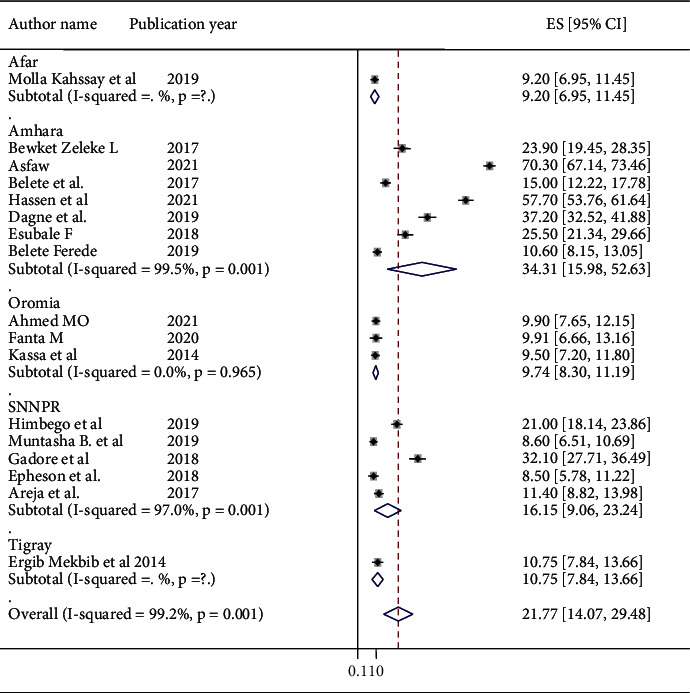
Forest plot displaying the pooled proportion of appropriate complementary feeding practices among mothers who had infants and young children aged between 6 and 24 months.

A total of seventeen primary studies were used to estimate the pooled proportion of ACFP in Ethiopia. The pooled proportion of ACFP among mothers who had IYC aged between 6 and 24 months was 21.77 (14.07, 29.48), and high heterogeneity was observed among primary studies conducted in Ethiopia (I^2^ = 99.2% and *P*=0.001) (Figure 2). Thus, the overall pooled proportion of ACFP among mothers who had IYC aged between 6 and 24 months was estimated by using a random-effects model.

### 6.3. Subgroup Analysis

Subgroup analysis was carried out based on region, mean of the sample size, publication year, and setting of study design to identify the possibility of heterogeneity. Heterogeneity was identified in all subgroup analyses. However, homogeneity was seen in the subgroup analysis of primary studies of Oromia region, and a fixed-effect model was used to estimate the pooled proportion of ACFP in Oromia Region. The subgroup analysis by region revealed that the highest pooled proportion of ACFP was in Amhara region [34.31 (15.98, 52.63)], and the lowest was in the Oromia region. However, the pooled proportion of Tigray and Afar regions was not presented due to one study being included in these regions [9.74 (8.3, 11.19)] (Figure 2).

The subgroup analysis of the pooled proportion of ACFP based on the mean sample size (553) showed that the pooled proportion of ACFP was identified to be higher among studies with a sample size of greater than 553 (22.28 with 95% CI: 11.00–33.55) compared to studies with a sample size of less than 553 (21.00 with 95% CI: 12.8–29.2). The pooled proportion of ACFP in the studies conducted after 2019 was greater (26.00 with 95% CI: 12.4–39.61) compared to the pooled proportion of ACFP in the studies conducted before 2019. Moreover, the pooled proportion of ACFP based on community-based versus facility-based cross-sectional study design indicated that the pooled proportion of ACFP was higher for facility-based cross-sectional studies (44.36 with 95% CI: 15.42–73.31) compared to community-based cross-sectional study design (16.89 with 95% CI: 11.56–22.22).

### 6.4. Publication Bias

**Figure 3:**
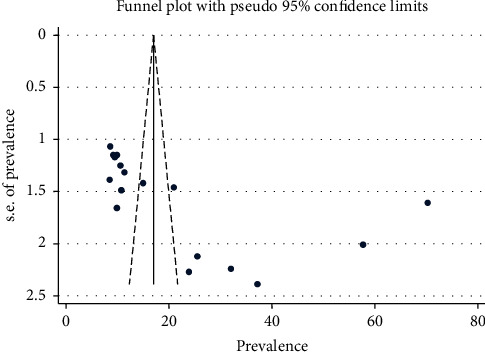
Funnel plot for examining publication bias of appropriate complementary feeding practices among mothers who had IYC aged between 6 and 24 months.

**Figure 4:**
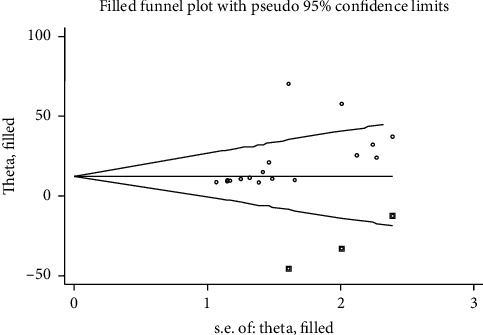
Funnel plots presenting the resulting trim and fill analysis for correcting publication bias. The circular dots indicate the included studies, and the rectangular dots display the missing studies imputed by the trim and fill method.

Publication bias among the 17 studies was examined using both funnel plots and Egger's regression test (Figure 3). The asymmetric shape shown by the funnel plot indicates the presence of publication bias among these studies. Egger's regression test also indicated the presence of publication bias across the studies (*P* value = 0.012). The Duval and Tweedie nonparametric trim and fill analysis was employed. Thus, publication bias was corrected when three studies were filled in the funnel plot by trim and fill analysis (Figure 4).

### 6.5. Factors Associated with Appropriate Complementary Feeding Practices

First, data extraction was conducted using a format prepared in the Microsoft Office Excel Worksheet. The heading of this form has headings for the same categories, and the odds ratios of associated factors with similar categories were extracted under a column. Finally, the studies that have associated factors with similar categories were included in the pooled analysis of OR for ACFP.

**Figure 5:**
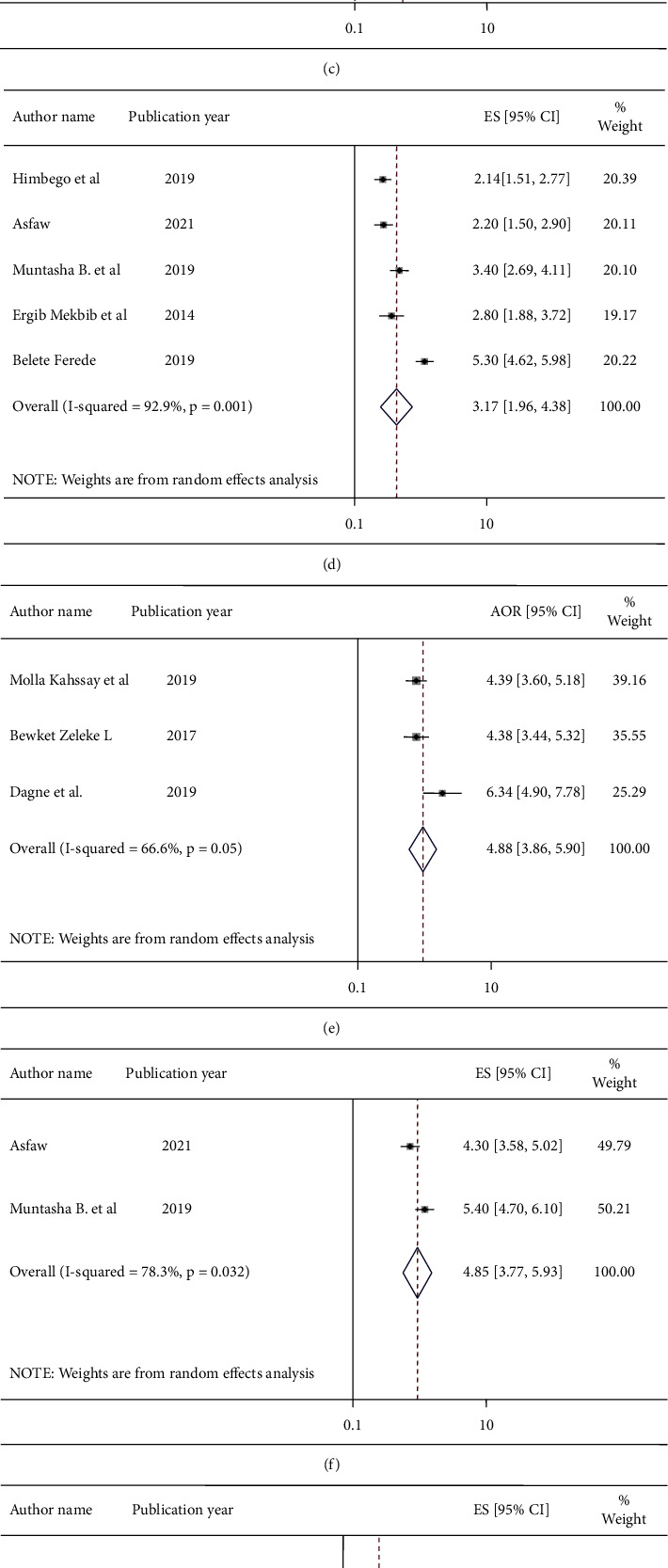
Forest plot displaying pooled odds ratios of the associated factors of appropriate complementary feeding practices (a) mothers' educational status of secondary school and above, (b) repeated antenatal care visits, (c) children aged between 12 and 24 months, (d) repeated postnatal care visits, (e) health education on IYC feeding practice, (f) knowledge of mothers on IYC feeding practice, (g) maternal age between 18 and 35 years, (h) institutional delivery, and (i) higher household wealth.

In this systematic review and meta-analysis, the overall pooled effect size of significantly associated factors of ACFP in this study were mothers' educational status of secondary school and above (OR = 3.36 with 95% CI: 3.03–3.69) [23, 24, 26, 27, 31], having repeated ANC visits (OR = 4.77 with 95% CI: 3.49–6.05) [18, 19, 26, 28], child's age between 12 and 24 months (OR = 3.7 with 95% CI: 2.75–4.65) [20, 23, 24, 27, 31], having repeated PNC visits (OR = 3.17 with 95% CI: 1.96–4.38) [16, 20, 21, 27, 31], having health education on IYC feeding practice (OR = 4.88 with 95% CI: 3.86–5.9) [17, 18, 26], knowledge of mothers on IYC feeding practice (OR = 4.85 with 95% CI: 3.77–5.93) [16, 21], maternal age between 18 and 35 years (AOR = 2.67 with 95% CI: 1.64–3.72) [20, 23, 25], institutional delivery (OR = 2.23 with 95% CI: 1.79–2.68) (18, 25), and higher household wealth (OR = 2.65 with 95% CI: 1.46–3.84) [23, 29] (Figure 5).

High heterogeneity was observed among primary studies reporting mothers' knowledge about IYC feeding practice, repeated ANC visits, children aged between 12 and 24 months, repeated PNC visits, higher household wealth, and maternal age between 18 and 35 years as significant associated factors of ACFP in Ethiopia. Moderate heterogeneity was seen among primary studies reporting mothers who had got health education on IYC feeding practice as significant associated factors of ACFP in Ethiopia. Low heterogeneity was observed among primary studies reporting mothers' educational status and institutional delivery as an associated factor, and the fixed-effect model was used to estimate the pooled odds ratio for studies with low heterogeneity. Moreover, a random-effects model was used for the studies with high and moderate heterogeneity (Figure 5).

## 7. Discussion

The pooled proportion of ACFP was determined, and the pooled predictors of ACFP were identified. To the best of our knowledge, this study is the first of its kind to estimate the pooled proportion of ACFP and its predictors. The national pooled proportion of ACFP among mothers who had IYC aged between 6 and 24 months in Ethiopia was found to be 21.77 (95% CI: 14.07, 29.48), and this finding coincided with the studies conducted in Bangladesh [13] and Ghana [39]. This could be due to the similarity in giving attention to ACFP at policymakers, health service manager, and health service provider level to encourage the means to increase mothers' practice of feeding IYCs aged between 6 and 24 months. This finding is higher than the study conducted in Malawi [5] and Ethiopia [14]. The possible explanation for the disparity between studies could be due to the study area, study period, socioeconomic status, and implementation of policies related to the IYC feeding practices of the country.

The subgroup analysis of the pooled proportion of ACFP indicated that the highest pooled proportion of ACFP was observed in Amhara region and the lowest pooled proportion of ACFP was observed in Oromia region. This disparity could be due to variations in socio-cultural and infrastructure between the regions. The disparity could also be explained as follows: (1) better family economic level or higher household wealth improves ACFP, (2) it is known that rural mothers are far from health facilities to get health services, and there are no transport facilities such as roads for rural mothers, (3) there are disparities in the socioeconomic level, access to health facilities, and infrastructure among regions, and (4) harmful traditional practices such as feeding eggs to children are taboo to the people in some rural areas.

Moreover, the studies conducted in Amhara region relatively covered different areas (about seven studies in different areas in Amhara region) compared to Oromia region (only three studies in two areas in the region). Thus, as the number of studies increases in the region, the strength of the pooled proportion of ACFP could increase due to an increase in the number of participants engaged in ACFP.

The pooled proportion of ACFP was higher among studies with a sample size greater than the mean of the total sample size of all included studies compared to studies with a sample size less than the mean of the total sample size of all included studies. The pooled proportion of ACFP was higher for facility-based cross-sectional studies compared to community-based cross-sectional studies. This might be due to better participants' experience of visiting health facilities to get information and engage in ACFP in facility-based studies than participants from community-based studies.

The pooled proportion of ACFP in the studies conducted after 2019 was greater than the pooled proportion of ACFP in the studies conducted before 2019. This could be because of an increase in maternal health service coverage such as ANC, PNC, and institutional delivery after 2019 [11]. The role of health extension workers who can implement health service packages during their home visits in the community could be responsible for the improvements in ANC, PNC, and delivery coverage. These health packages include ANC, PNC, and institutional delivery services such as advice about pregnancy danger signs, danger signs during the postpartum period, IYC feeding practices, and encouraging institutional delivery. The health service providers in the maternal health service outlets in all health facilities are also working together with health extension workers to improve the health services. Moreover, IYC feeding advice is a component of ANC, PNC, and delivery services. Thus, there is a possible impact on ACFP.

The findings of this systematic review and meta-analysis indicated that the pooled odds of ACFP among mothers who had the educational status of secondary school and above increased compared to the four primary studies out of six included studies in the subgroup analysis. Moreover, the confidence interval of the odds ratio of mothers' educational status of secondary school and above decreased compared to the six primary studies included in the subgroup analysis. Thus, the odds of ACFP were modified, and ACFP was higher among mothers who had the educational status of secondary school and above than mothers who had lower educational status. Previous studies also indicated that mothers who had the educational status of secondary school and above had better ACFP [14, 40–44]. This could be due to mothers' higher educational status increasing their knowledge of ACFP, and their educational status empowers mothers to decide on their IYC feeding practices. It is also known that mothers' higher educational status increases the opportunity of getting a better job and higher household wealth to increase ACFP.

In this study, the odds ratio of repeated ANC visits in this systematic review and meta-analysis increased compared to only one study from the four studies included in the subgroup analysis, and the confidence interval of the odds ratio of repeated ANC visits narrowed compared to only one study out of four studies included in the subgroup analysis. The odds ratio of repeated PNC visits increased compared to three studies out of five studies involved in the subgroup analysis. The odds ratio of mothers who had institutional delivery also increased compared to one study out of the two studies involved in the subgroup analysis. The confidence interval of the odds ratio of mothers who had institutional delivery narrowed compared to one study out of two studies included in the subgroup analysis. Therefore, the odds of ACFP increased, and mothers who had repeated ANC visits, repeated PNC visits, and institutional delivery had more ACFP than mothers who did not have these. Different studies also revealed that mothers who had repeated ANC visits, repeated PNC visits, and institutional delivery increased ACFP [41–43, 45–47]. This might be because IYC feeding advice and counseling are components of ANC, PNC, and delivery services. Besides, it is clear that mothers who have ANC, PNC, and institutional delivery could have the opportunity to get knowledge of ACFP.

The confidence interval of the odds ratio of health education on IYC feeding practice was reduced compared to one study out of three studies included in the subgroup analysis of the current study. The odds ratio of health education on IYC feeding practice enlarged equated to two studies out of three studies included in the subgroup analysis. Similarly, the odds ratio of knowledge of mothers on IYC feeding practice increased compared to one study out of two studies. Hence, the odds of ACFP were boosted, and health education and knowledge of mothers on IYC feeding practice had a place to improve ACFP in the current study. Similar studies also showed that health education and knowledge of mothers on IYC feeding practice increased ACFP [40, 44]. Health education increases the knowledge of mothers that can help mothers to overcome IYC feeding difficulties or challenges. Mothers' knowledge of ACFP can be basic to include all components of nutrition for the child during feeding.

The odds ratio of mothers who had infants aged between 12 and 24 months increased compared to the two studies out of five studies included in the subgroup analysis, and the confidence interval of the odds ratio for those mothers thinned compared to one study out of five studies considered in the subgroup analysis. Therefore, the odds of ACFP among mothers who had infants aged between 12 and 24 months were increased, and this systematic review and meta-analysis strengthened that mothers who had infants aged between 12 and 24 months had better ACFP than other participants who had children aged less than 12 months. The same findings in other studies indicated that mothers who had infants aged between 12 and 24 months enhanced ACFP [14, 46, 48]. Culturally, mothers believe that young babies aged less than 12 months could not be able to digest food such as eggs, meat, and vegetables before the age of one year. Moreover, some of the mothers could not commence complementary feeding at the infant age of six months.

The odds ratio of maternal age between 18 and 35 years increased compared to two studies from the three studies included in the subgroup analysis. Thus, this study strengthened that mothers aged between 18 and 35 years had more ACFP than other participants aged greater than 35 years or less than 18 years. The findings of other studies also identified that mothers aged between 18 and 35 years improved ACFP [48]. Younger mothers aged less than 35 years and greater than 18 years have a better experience and educational status than older and teenage mothers to trigger ACFP by encouraging different types of food. Unwanted pregnancies could be more common among mothers aged less than 18 years and greater than 35 years. Thus, mothers might neglect to feed a child appropriately.

The odds ratio of higher household wealth increased compared to one study from the two studies involved in the subgroup analysis. Therefore, mothers who had higher household wealth had better ACFP compared to mothers who had lower household wealth. Other studies support this finding [42, 43, 45–47, 49]. Mothers who have higher household wealth are more likely to have food security for their babies. Thus, these households can afford to get different food items that can fulfill appropriate IYC feeding practices. These mothers could also have better opportunities to get maternal health care services such as ANC, PNC, and institutional delivery.

### 7.1. Limitations

The limitation of this systematic review and meta-analysis was that only five regions from nine regions of Ethiopia were included in this study due to a limited number of studies. Small numbers of studies were included to analyze the pooled effect of some significantly associated factors of ACFP. However, the strength of this study was that both published and unpublished studies were included.

## 8. Conclusions

The pooled proportion of ACFP among mothers who had IYC aged between 6 and 24 months was low in Ethiopia. Mother's educational status of secondary school and above, repeated ANC visits, child's age between 12 and 24 months, repeated PNC visits, health education on IYC feeding practice, knowledge of mothers on IYC feeding practice, maternal age between 18 and 35 years, institutional delivery, and higher household wealth were independent predictors of ACFP of mothers who had IYC aged between 6 and 24 months. Therefore, intervention is crucial based on predictors of ACFP, and integrated interventions such as health education and increasing maternal health service uptake are also still required to improve appropriate complementary feeding practices. Moreover, more intervention is important for mothers who have children aged less than 12 months, mothers aged above 35 years and less than 18 years, lower mothers' educational status, and lower household wealth.

## Figures and Tables

**Table 1:** Characteristics of included studies presenting the proportion of appropriate complementary feeding of mothers who had infants and young children aged between 6 and 24 months in Ethiopia.

Authors	Publication year	Region	Study area	Study design	Sample size	Cases of ACFP	Prevalence	Quality score
Molla Kahssay et al. [20]	2019	Afar	Assayita district	Cross-sectional	632	57	9.2	8
Bewket Zeleke [19]	2017	Amhara	Feres Bet district	Cross-sectional	353	84	23.9	7
Ahmed MO [21]	2021	Oromia	Gemachis	Cross-sectional	6hh74	65	9.9	8
Himbego et al. [22]	2019	SNNPR	Kedida Gamela	Cross-sectional	777	154	21	8
Asfaw [18]	2021	Amhara	Debre Birhan town	Cross-sectional	805	551	70.3	8
Muntasha et al. [23]	2019	SNNPR	Bensa district	Cross-sectional	688	58	8.6	8
Gadore et al. [24]	2018	SNNPR	Gibe district	Cross-sectional	434	134	32.1	8
Belete et al. [25]	2017	Amhara	Debre Markos district	Cross-sectional	634	91	15	8
Fanta [32]	2020	Oromia	Horro district	Cross-sectional	325	32	9.91	7
Kassa et al. [26]	2014	Oromia	Arsi Negele district	Cross-sectional	626	58	9.5	8
Epheson et al. [17]	2018	SNNPR	Damot Weyde district	Cross-sectional	404	34	8.5	7
Hassen et al. [27]	2021	Amhara	Kalu district	Cross-sectional	605	340	57.7	8
Dagne et al. [28]	2019	Amhara	Debre Tabor Hospital	Cross-sectional	409	152	37.2	8
Ergib Mekbib et al. [29]	2014	Tigray	Abyi Adi	Cross-sectional	434	46	10.75	8
Esubalew [31]	2018	Amhara	Hospitals	Cross-sectional	422	107	25.5	7
Belete Ferede [33]	2019	Amhara	Faggeta Lekoma district	Cross-sectional	605	63	10.6	8
Areja et al. [30]	2017	SNNPR	Damot Sore district	Cross-sectional	582	62	11.4	8

## Data Availability

The datasets used and analyzed during the current study are available from the corresponding author upon reasonable request.

## Supplementary Materials

Supplementary Materials S1 Supplementary Materials: the search terms were used to get all articles related to appropriate complementary feeding practice and its associated factors among mothers who have children aged between 6 and 24 months in Ethiopia.
