# Supplementary material for: Appropriate Complementary Feeding Practice and Its Associated Factors among Mothers Who Have Children Aged between 6 and 24 Months in Ethiopia: Systematic Review and Meta-Analysis
Source: J Nutr Metab. 2022 Sep 22;2022:1548390. doi: 10.1155/2022/1548390 (PMC9553750; doi:10.1155/2022/1548390)
Supplement: Supplementary Materials — S1 Supplementary Materials: the search terms were used to get all articles related to appropriate complementary feeding practice and its associated factors among mothers who have children aged between 6 and 24 months in Ethiopia. [file 1548390.f1.docx]

**S_1_ Supplementary Material_._ The search terms used to identify articles related to “Appropriate complementary feeding practice and its associated factors among mothers who have children aged between 6 and 24 months in Ethiopia”.**

("epidemiology"[Subheading] OR "epidemiology"[All Fields] OR "prevalence"[All Fields] OR "prevalence"[MeSH Terms]) OR magnitude[All Fields] AND (appropriate[All Fields] AND ("infant nutritional physiological phenomena"[MeSH Terms] OR ("infant"[All Fields] AND "nutritional"[All Fields] AND "physiological"[All Fields] AND "phenomena"[All Fields]) OR "infant nutritional physiological phenomena"[All Fields] OR ("complementary"[All Fields] AND "feeding"[All Fields]) OR "complementary feeding"[All Fields]) AND "practice"[All Fields] AND associated[All Fields] AND factors[All Fields]) OR (appropriate[All Fields] AND feeding[All Fields] AND "practice"[All Fields] AND associated[All Fields] AND factors[All Fields]) OR (appropriate[All Fields] AND ("weaning"[MeSH Terms] OR "weaning"[All Fields]) AND "practice"[All Fields] AND associated[All Fields] AND factors[All Fields]) OR (optimum[All Fields] AND ("infant nutritional physiological phenomena"[MeSH Terms] OR ("infant"[All Fields] AND "nutritional"[All Fields] AND "physiological"[All Fields] AND "phenomena"[All Fields]) OR "infant nutritional physiological phenomena"[All Fields] OR ("complementary"[All Fields] AND "feeding"[All Fields]) OR "complementary feeding"[All Fields]) AND "practice"[All Fields] AND associated[All Fields] AND factors[All Fields]) OR (appropriate[All Fields] AND uptake[All Fields] AND minimum[All Fields] AND acceptable[All Fields] AND ("diet"[MeSH Terms] OR "diet"[All Fields]) AND associated[All Fields] AND factors[All Fields]) AND (("mothers"[MeSH Terms] OR "mothers"[All Fields]) AND who[All Fields] AND ("child"[MeSH Terms] OR "child"[All Fields] OR "children"[All Fields]) AND ("aged"[MeSH Terms] OR "aged"[All Fields]) AND 6-23[All Fields] AND months[All Fields] AND ("ethiopia"[MeSH Terms] OR "ethiopia"[All Fields]))
